# Scalable Statistical Channel Estimation and Its Applications in User-Centric Cell-Free Massive MIMO Systems

**DOI:** 10.3390/s25113263

**Published:** 2025-05-22

**Authors:** Ling Xing, Dongle Wang, Xiaohui Zhang, Honghai Wu, Kaikai Deng

**Affiliations:** School of Information Engineering, Henan University of Science and Technology, Luoyang 471023, China; xingling_my@haust.edu.cn (L.X.); keren@ldy.edu.rs (D.W.); honghai2018@haust.edu.cn (H.W.); dengkk24@haust.edu.cn (K.D.)

**Keywords:** cell-free, massive MIMO, scalability, spectral efficiency, channel state information, power control

## Abstract

Cell-free massive multiple-input multiple-output (mMIMO) technology utilizes collaborative signal processing to significantly improve system performance. In cell-free mMIMO systems, accurate channel state information (CSI) is a key element in improving the overall system performance. The existing statistical CSI acquisition methods for large-scale fading (LSF) processing schemes assume that each access points (APs) provides service to all user equipments (UEs) in the system. However, as the number of UEs or APs increases, the computational complexity of statistical CSI estimation tends to infinity, which is not scalable in large-scale networks. To address this limitation, this paper proposes a scalable statistical CSI estimation method under the user-centric cell-free mMIMO system, which blindly estimates the partial statistical CSI required for LSF schemes using uplink (UL) data signals. Additionally, the estimated partial statistical CSI can also be used for downlink (DL) LSF precoding (LSFP) or power control in fully distributed precoding. Simulation results show that under the LSFP scheme, the proposed method can achieve comparable spectral efficiency (SE) with the traditional CSI acquisition scheme while ensuring scalability. When applied to power control in fully distributed precoding, it significantly reduces the fronthaul link CSI overhead while maintaining a nearly similar SE performance compared to existing solutions.

## 1. Introduction

Cell-free mMIMO has gained significant attention due to its ability to provide universally good service to UEs, and it is regarded as a key technology for driving the development of 6G in the future. Traditional cellular mMIMO, which relies on cell division, often results in poor signal quality for edge UEs, while spectral efficiency progressively declines as the cell coverage area expands. In contrast, in a cell-free mMIMO system, numerous distributed APs are deployed near the UEs, with each AP equipped with a small number of antennas. All APs are connected to a central processing unit (CPU) responsible for handling all signals via wired or wireless fronthaul links. The APs collaborate to coherently transmit/receive signals for the UE, thereby reducing the interference issues faced by users at the cell edge. Since each UE is mainly influenced by the signals from nearby APs, this cell-free mMIMO system composed of numerous APs is also referred to as a user-centric network. This system architecture fully leverages the advantages of macro diversity, enhancing the overall system SE and ensuring a uniform quality of service for UEs [1,2].

As research deepens, the acquisition and optimization of CSI have become increasingly critical in cell-free mMIMO systems. The performance of such systems heavily depends on accurate CSI estimation. However, existing CSI estimation methods often suffer from high computational complexity and poor scalability in large-scale networks. In large systems, traditional CSI acquisition techniques typically involve extensive data exchange and complex computations, which consume substantial computational resources and degrade real-time processing capabilities, thereby limiting system performance. Particularly in uncertain channel environments, the robustness and efficiency of beamforming algorithms face significant challenges [3,4]. Therefore, there is an urgent need for new methods to address the complexity and scalability issues in CSI acquisition, thereby enhancing the adaptability and intelligence of the overall system [5].

### 1.1. Related Work

In cell-free mMIMO systems, CSI is crucial for optimizing system performance. The system can generally be categorized into three types based on the level of collaboration between APs as folllows: distributed [6,7], centralized [8,9,10], and semi-distributed large-scale fading (LSF) processing [11]. In a centralized system, the instantaneous CSI of the whole network is collected at the CPU, which is used to suppress interference among the UEs. In [9], the authors designed a near-optimal max–min fairness (MMF) power allocation method for centralized zero-forcing (ZF) precoding by iteratively maximizing the minimum SE of all UEs, and they combined this with a low complexity heuristic scheme. In [12], the author proposed a centralized minimum mean square error (MMSE) precoding scheme and its joint power optimization strategy. The author proved that the centralized MMSE precoding can provide higher SE than centralized ZF precoding. Although the centralized scheme can effectively suppress inter-user interference and improve spectral efficiency, it relies on the CPU to conduct the signal processing and channel estimation of the entire network. In contrast, distributed techniques offload the signal processing task to distributed APs by using local CSI. In [13], full-pilot ZF (FZF) precoding was proposed to eliminate the interference between UEs using local CSI at the APs, but the actual performance of the system is determined by the quantity of antenna arrays at each AP. To alleviate this constraint, local partial ZF (LP-ZF) precoding was proposed in [14] by allowing each AP to reduce its interference to the strongest UEs. In [10], the authors proposed a local partial MMSE (LP-MMSE) precoding to suppress inter-UE interference using UL-DL duality at the APs, which greatly improves the system performance, and this scheme had no requirement on the number of antennas at the APs. Compared to FZF and LP-ZF, LP-MMSE offers greater flexibility. Although computing local CSI distributively at each AP can avoid the fronthaul consumption of sending local CSI to the CPU, the performance of the distributed approaches is far inferior to the centralized methods.

To reduce the performance gap between distributed and centralized schemes, researchers have proposed utilizing LSF processing techniques to enhance the performance of distributed solutions [15,16]. During LSF processing, the LSF weight calculations require network-wide statistical CSI at the CPU. Previous studies on LSF generally assumed perfect instantaneous CSI to obtain the required statistical CSI for LSF weights’ calculation [11], but this is only an ideal state and is not feasible in practical applications. In [17], the authors proposed computing statistical CSI by performing a weighted average of local channel estimates from each AP, which requires statistical CSI exchange between APs and CPU via fronthaul links. In [18], a new method was proposed for estimating CSI at the CPU using uplink data information. Although this method can reduce the fronthaul CSI overhead introduced during the LSF processing, it was derived under the condition that all APs provide services to all UEs. The complexity of statistical CSI estimation for each UE gradually increases as the number of UEs keeps increasing. Thus, this method is not scalable to large-scale networks with numerous UEs. In addition, the authors in [19] designed a distributed fractional power allocation (FPA-dis) strategy that relies on large-scale fading factors for maximum ratio (MR) precoding, and in the literature [20], a generalized fractional power control (GFPA) algorithm was further proposed to extend it to other precoding schemes. However, the GFPA algorithm requires local CSI to be sent to the CPU for power control, which leads to significant statistical CSI overhead on the fronthaul links.

### 1.2. Contributions

In this research, we propose a scalable method to estimate the statistical CSI in a user-centric cell-free mMIMO system, where each AP only serves a subset of UEs within the system. The proposed method uses uplink data to blindly estimate the partial statistical CSI required for LSF processing or power control in fully distributed DL beamforming. It avoids the statistical CSI exchange between APs and CPU for LSF processing and distributed power control, thus significantly reducing the statistical CSI overhead on the fronthaul link while ensuring system scalability. The key contributions can be summarized as follows:

- Under the user-centric model, we propose a method that utilizes UL data signals to blindly estimate the partial statistical CSI necessary for LSF schemes. This method can achieve spectral efficiency comparable to, or even better than, that of conventional schemes while being scalable to large-scale networks with numerous UEs.
- We show that the scalable CSI estimation method proposed in this paper can also be used for distributed power control methods such as GFPA [20]. By blindly estimating the required CSI using UL data, this approach attains approximately the same SE as the traditional full statistical CSI feedback, which significantly reduces the fronthaul overhead for CSI transmission.
- The proposed power control and statistical channel estimation methods are scalable to large-scale networks with numerous UEs, as each AP only provides services for a subset of UEs, with limited computational complexity in a user-centric framework.

*Notation:* ${\left(\cdot \right)}^{*}$ denotes conjugate; ${\left(\cdot \right)}^{T}$ denotes transpose; ${\left(\cdot \right)}^{H}$ denotes conjugate transpose; ${∥\cdot ∥}_{2}$ denotes Euclidean norm; the expectation operator is indicated by E{·}; $\mathrm{diag}\left(\cdot \right)$ denotes the diagonal matrix; $0_{m\times n}$ denotes an *m*-row *n*-column zero matrix; and $I_{N}$ is a identity matrix with *N* rows and *N* columns. $N_{C}\left(\cdot \right)$ denotes Complex Gaussian Distribution.

## 2. System Model

**Figure 1:**
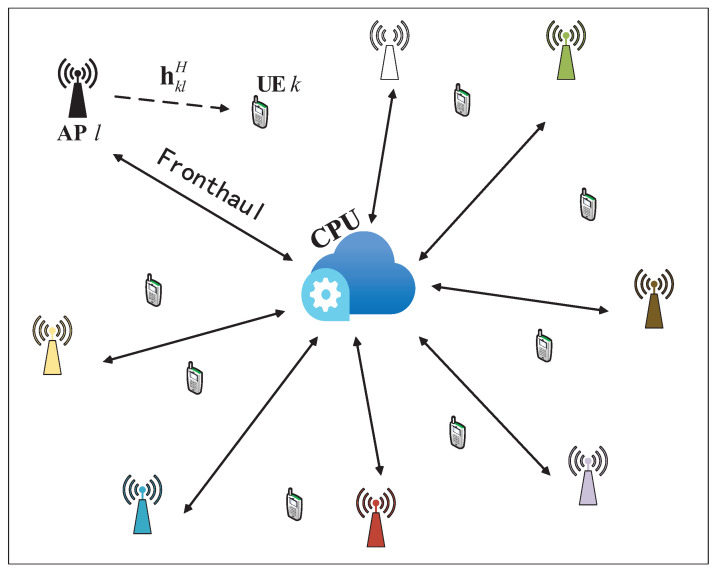
Cell-free mMIMO network with numerous APs and UEs.

We consider a cell-free mMIMO system, as illustrated in Figure 1, consisting of *K* single-antenna UEs and *L* APs that are randomly deployed across a specific area. Each AP is equipped with *N* antennas, where M=NL and M≫K. Each AP connects to the CPU via a fronthaul link, allowing them to share CSI and data between them via the CPU. We choose the conventional time division duplex (TDD) operating mode [10,21]. Each channel coherence block is segmented into channel uses allocated for UL and DL communications. Specifically, $\tau_{p}$ channel uses are assigned for channel estimation, $\tau_{u}$ channel uses for UL data transmission, and $\tau_{d}$ channel uses will be used for DL data transmission. The channel between AP *l* and UE *k* is represented by $h_{kl}\in C^{N}$, and satisfies the following:

(1) $$\begin{array}{c} h_{kl}∼N_{C}\left(0,R_{kl}\right), \end{array}$$

where $R_{kl}=E\left\{h_{kl}h_{kl}^{H}\right\}$ is a matrix describing the correlation of channels between AP *l* and UE *k* at different spatial locations. ${\hat{h}}_{kl}$ denotes the MMSE estimation of the channel $h_{kl}$, and ${\tilde{h}}_{kl}=h_{kl}-{\hat{h}}_{kl}$ is the error generated during the estimation process and satisfies the following:

(2) $$\begin{array}{c} {\tilde{h}}_{kl}∼N_{C}\left(0,C_{kl}\right), \end{array}$$

where $C_{kl}=E\left\{{\tilde{h}}_{kl}{\tilde{h}}_{kl}^{H}\right\}$ is a matrix describing the covariance relationship between the channel estimation errors ${\tilde{h}}_{kl}$.

To enable system scalability, we use the user-centric dynamic cooperative clustering (DCC) framework from [10]. In this framework, each UE is served by a subset of APs that are chosen according to the best channel conditions. The set of APs serving UE *k* is denoted by $M_{k}\subset \left\{1,\cdots ,L\right\}$, which is a subset of all *L* APs. Conversely, each AP *l* serves a set of UEs $P_{l}\subset \left\{1,\cdots ,K\right\}$. We establish the service relationship between UE *i* and AP *l* through the diagonal service association matrix $D_{il}\in C^{N\times N}$. We assume that if an AP is determined to serve a particular UE, it will utilize all of its available antennas for that UE. The value of the diagonal matrix $D_{il}$ is determined as follows: If $i\in P_{l}$, then $D_{il}=I_{N}$; otherwise, $D_{il}=0_{N}$. Let diagonal matrix $D_{k}=\mathrm{diag}\left(D_{k1},\ldots ,D_{kL}\right)C^{M\times M}$ represent the service relationships between all APs and UE *k*.

## 3. Uplink Data Transmission and Scalable Statistical CSI Estimation

In this section, we first introduce the LSF detection (LSFD) scheme for UL transmission and then propose a scalable approach for the blind estimation of the partial statistical CSI required for LSF schemes based on UL data signals. LSFD consists of two layers of data decoding processes, where local channel information at each AP is utilized for the first layer of local decoding. The signal received at AP *l* can be formulated as follows:

(3) $$r_{l}=\sum_{k=1}^{K}h_{kl}s_{k}+n_{l},\;1\leq l\leq L,$$

where $s_{k}$ represents the signal transmitted by UE *k*, with signals from different UEs being mutually independent, and $n_{l}$ is the additive noise at AP *l*.

After AP *l* integrates the data signals from all serving UEs, it processes these signals according to the local combining vectors $v_{il},\;i=1,\ldots ,K$ to generate a preliminary estimate of the uplink signal. The local signal for UE *k* is estimated as follows:

(4) $${\hat{s}}_{kl}=v_{kl}^{H}D_{kl}r_{l}=v_{kl}^{H}D_{kl}h_{kl}s_{k}+\sum_{i=1,i\neq k}^{K}v_{kl}^{H}D_{il}h_{il}s_{i}+v_{kl}^{H}n_{l}.$$

Following the local combination process, each AP *l* sends the estimated local signal values $\left\{{\hat{s}}_{il}:i=1,\ldots K\right\}$ to the CPU.

Upon obtaining the transmitted signal from each AP, the CPU will use the network-wide statistical CSI to generate the LSFD vector for the second layer decoding of each UE. The LSFD vector that CPU selected for UE *k* is denoted by $e_{k}={\left[e_{k1},\ldots ,e_{kL}\right]}^{T}$. The CPU’s final estimation of the uplink data for UE *k* is as follows:

(5) $${\hat{s}}_{k}=\sum_{l=1}^{L}e_{kl}^{*}{\hat{s}}_{kl}=\sum_{l=1}^{L}e_{kl}^{*}v_{kl}^{H}D_{kl}r_{l}.$$

The LSFD vector’s basic criteria are defined in [11] as follows:

(6) $$e_{k}={\left(\sum_{i=1}^{K}p_{i}E\left\{g_{ki}g_{ki}^{H}\right\}+\sigma^{2}\Psi_{k}\right)}^{-1}E\left\{g_{kk}\right\},$$

where

(7) $$g_{ki}={\left[v_{k1}^{H}h_{i1},\ldots ,v_{kL}^{H}h_{iL}\right]}^{T},$$

(8) $$\Psi_{k}=\mathrm{diag}\left(E\left\{{∥v_{k1}∥}_{2}^{2}\right\},\ldots ,E\left\{{∥v_{kL}∥}_{2}^{2}\right\}\right).$$

$p_{i}$ is the power of UE *i* during UL data transmission. However, Equation (6) is derived under the assumption that every AP serves all UEs. As the quantity of UEs increases, the computational complexity of this method tends to approach infinity. In reference [18], a CSI estimation (CSIE) method is proposed based on Equation (6). However, this method also apparently fails to meet the requirements of scalability.

Under the user-centric DCC framework, the scalable LSFD vector of UE *k* can be obtained by the following equation:

(9) $${\tilde{e}}_{k}={\left(\sum_{i\in L_{k}}p_{i}E\left\{q_{ki}q_{ki}^{H}\right\}+\sigma^{2}\theta_{k}\right)}^{-1}E\left\{q_{kk}\right\},$$

where $L_{k}=\left\{i:D_{i}D_{k}\neq 0_{LN}\right\}$ is defined as the cluster of UEs that share the same APs with UE *k*. $\theta_{k}=\mathrm{diag}\left(E\left\{{∥v_{k1}D_{k1}∥}_{2}^{2}\right\},\ldots ,E\left\{{∥v_{kL}D_{kL}∥}_{2}^{2}\right\}\right)$. In particular, note that

(10) $$q_{ki}=\left[\begin{array}{c} v_{k1}^{H}D_{k1}h_{i1}\\ \vdots \\ v_{kL}^{H}D_{kL}h_{iL} \end{array}\right]$$

represents the combination of the combining vectors of UE *k* with the channel vectors of UE *i*. $q_{ki}$ is an *L*-dimensional sparse vector, with an effective dimension of $\left|M_{k}\right|$ ($\left|M_{k}\right|$ denotes the cardinality of the set $M_{k}$); $\theta_{k}$ is a sparse diagonal matrix, since when $l\notin M_{k}$, the matrix $D_{kl}=0_{N}$; and the corresponding entries in $q_{ki}$ and $\theta_{k}$ are zero. The all-zero columns/rows in $q_{ki}$ and $\theta_{k}$ will be removed before the matrix inversion is taken. So Equation (9) only needs to compute the inverse of a $\left|M_{k}\right|\times \left|M_{k}\right|$ matrix, while Equation (6) needs to compute the inverse of a L×L matrix. Therefore, the computational complexity of Equation (9) is much lower compared to Equation (6).

### Scalable Statistical CSI Estimation

From Equation (9), it can be seen that when using the traditional method to compute ${\tilde{e}}_{k}$, the CPU needs to compute the complex matrix $E\{q_{ki}q_{ki}^{H}\}$ and the complex vector $E\{q_{kk}\}$, and this requires each AP to send its locally estimated statistical CSI to the CPU via fronthaul links, which lead to CSI overhead in fronthaul. However, we can utilize the estimated UL signals from all APs, which are sent to the CPU, as shown in Equation (4), as training data to blindly estimate ${\tilde{e}}_{k}$ at the CPU. Let $F_{k}=\sum_{i\in L_{k}}p_{i}E\left\{q_{ki}q_{ki}^{H}\right\}+\sigma^{2}\theta_{k}$, $f_{k}=E\left\{q_{kk}\right\}$ represent the partial statistical CSI needed to compute the LSFD vector in Equation (9).

Let $z={\left[s_{1},\ldots ,s_{K}\right]}^{T}$ be the collective data signal from all UEs and ${\vec{z}}_{k}={\left[{\hat{s}}_{k1},\ldots ,{\hat{s}}_{kL}\right]}^{T}$ represent the estimated signal for UE *k*. While all UEs transmit their signals, only the APs corresponding to $l\in M_{k}$ will actually estimate them. Thus, we have the following:

(11) $${\vec{z}}_{k}=G_{k}z+{\vec{n}}_{k},$$

where

(12) $$G_{k}=\left[\begin{array}{ccc} v_{k1}^{H}D_{k1}h_{11} & \ldots & v_{k1}^{H}D_{k1}h_{K1}\\ \vdots & \ddots & \vdots \\ v_{kL}^{H}D_{kL}h_{1L} & \ldots & v_{kL}^{H}D_{kL}h_{KL} \end{array}\right]$$

represents the equivalent channel matrix of UE *k*, which is a L×K sparse matrix, with corresponding element $v_{kl}^{H}D_{kl}h_{il}=0$, when $l\notin M_{k}$. ${\vec{n}}_{k}={\left[\begin{array}{c} v_{k1}^{H}D_{k1}n_{1},\ldots ,v_{kL}^{H}D_{kL}n_{L} \end{array}\right]}^{T}$ is a sparse noise vector. Considering that the transmission signals of different UEs are independent, we have the following:

(13) $$\begin{array}{cc} E\left\{{\vec{z}}_{k}{\vec{z}}_{k}^{H}\right\} & =E\left\{\left(G_{k}z+{\vec{n}}_{k}\right){\left(G_{k}z+{\vec{n}}_{k}\right)}^{H}\right\}\\ & =E\left\{G_{k}zz^{H}G_{k}^{H}\right\}+E\left\{{\vec{n}}_{k}{\vec{n}}_{k}^{H}\right\}\\ & =\sum_{i\in L_{k}}p_{i}E\left\{q_{ki}q_{ki}^{H}\right\}+\sigma^{2}\theta_{k} \end{array}$$

The correlation between the original transmission signal $s_{k}$ from UE *k* and its local estimate ${\hat{s}}_{kl}$, as illustrated in Equation (4), can be written as follows:

(14) $$\begin{array}{cc} E\{{\hat{s}}_{kl}s_{k}^{*}\} & =E\left\{v_{kl}^{H}D_{kl}h_{kl}s_{k}s_{k}^{*}\right\}\;+E\left\{\sum_{i=1,i\neq k}^{K}v_{kl}^{H}D_{il}h_{il}s_{i}s_{k}^{*}\right\}\;+E\left\{v_{kl}^{H}n_{l}s_{k}^{*}\right\}\\ \; & =p_{k}E\left\{v_{kl}^{H}D_{kl}h_{kl}\right\}. \end{array}$$

Thus, we have:

(15) $$F_{k}=E\left\{{\vec{z}}_{k}{\vec{z}}_{k}^{H}\right\},$$

(16) $$f_{k}=E\left\{q_{kk}\right\}={\left[\frac{1}{p_{k}}E\left\{{\hat{s}}_{k1}s_{k}^{*}\right\},\ldots ,\frac{1}{p_{k}}E\left\{{\hat{s}}_{kL}s_{k}^{*}\right\}\right]}^{T}.$$

During the *c*th coherent block, when utilizing channel *t*, we define ${\vec{z}}_{k}^{tc}$ as the local estimation of the uplink data signal for UE *k*. Throughout the entire coherent block, $Z_{k}^{c}=\left[{\vec{z}}_{k}^{lc},\ldots ,{\vec{z}}_{k}^{\tau_{u}c}\right]$ is defined as the overall estimation of the uplink data signal for UE *k*. Then, $F_{k}$ can be estimated as follows:

(17) $${\hat{F}}_{k}=\frac{1}{\tau_{u}C}\sum_{c=1}^{C}Z_{k}^{c}{\left(Z_{k}^{c}\right)}^{H},$$

where *C* denotes the total number of coherent blocks.

We found that to calculate $f_{k}$, we need the actual UL data signal $s_{k}$ sent by UE *k*, which is unavailable to the CPU. However, the CPU can integrate the local estimation of UE *k*’s transmitted signal at each AP in Equation (4) to estimate $s_{k}$, which is given as follows:

(18) $${\tilde{s}}_{k}=\sum_{l\in M_{k}}{\hat{s}}_{kl}.$$

Then, the CPU makes a decision on ${\tilde{s}}_{k}$ to obtain the preliminary detection result ${\ddot{s}}_{k}$ of the uplink data signal for UE *k*. If the estimation error of UE *k* at each AP is small, we can perfectly replace $s_{k}$ with ${\ddot{s}}_{k}$, and we have the following:

(19) $$f_{k}\approx {\left[\frac{1}{p_{k}}E\left\{{\hat{s}}_{k1}{\ddot{s}}_{k}^{*}\right\},\ldots ,\frac{1}{p_{k}}E\left\{{\hat{s}}_{kL}{\ddot{s}}_{k}^{*}\right\}\right]}^{T}.$$

Let ${\ddot{s}}_{k}^{tc}$ be the overall estimation of the UL data signal for UE *k* at the *t*th channel use during the *c*th coherent block. In addition, let ${\hat{s}}_{kl}^{tc}$ represent the local estimation of the uplink signal from UE *k* at AP *l* during the *t*th channel use of the *c*th coherent block. Across all channel uses in the cth coherence block, we have ${\ddot{z}}_{k}^{c}=\left[{\ddot{s}}_{k}^{lc},\ldots ,{\ddot{s}}_{k}^{\tau_{u}c}\right]$ and $\left.{\hat{z}}_{kl}^{c}=\left[{\hat{s}}_{kl}^{lc},\ldots ,{\hat{s}}_{kl}^{\tau_{u}c}\right]\right.$. Then, $f_{k}$ can be expressed as follows:

(20) $${\hat{f}}_{k}=\frac{1}{p_{k}\tau_{u}C}{\left[\sum_{c=1}^{C}{\hat{z}}_{kl}^{c}{\left({\ddot{z}}_{k}^{c}\right)}^{H},\ldots ,\sum_{c=1}^{C}{\hat{z}}_{kL}^{c}{\left({\ddot{z}}_{k}^{c}\right)}^{H}\right]}^{T}.$$

From Equation (17) and Equation (20), we can directly estimate the LSFD vector ${\tilde{e}}_{k}$ for UE *k* as follows:

(21) $${\tilde{e}}_{k}={\hat{F}}_{k}^{-1}{\hat{f}}_{k}.$$

## 4. Downlink Transmission Using Estimated Statistical CSI

In the process of downlink data transmission, the transmitted signal from AP *l*, denoted as $x_{l}$, is a superposition of individually precoded signals intended for each UE served by that AP, which is given as follows:

(22) $$x_{l}=\sum_{i=1}^{K}D_{il}w_{il}\varsigma_{i}=\sum_{i\in P_{l}}w_{il}\varsigma_{i},$$

where $\left\{\varsigma_{i}:i=1,\ldots ,K\right\}$ are mutually independent unit power data symbols sent to each UE during downlink transmission, and they satisfy $E\left\{{∥\varsigma_{k}∥}_{2}^{2}\right\}=1$ for any UE *k*. $w_{il}\in C^{N}$ denotes the effective precoding vector assigned to UE *i* by AP *l*, which contains the joint effect of precoding and power control. In the user-centric DCC framework, the effective transmit precoding vector is defined as follows:

(23) $$D_{il}w_{il}=\left\{\begin{array}{cc} w_{il} & \mathrm{if}\;l\in M_{i}\\ 0_{N} & \mathrm{if}\;l\notin M_{i} \end{array}\right..$$

The choice of precoding vectors should be satisfied at each AP *l* as follows:

(24) $$E\{\|x_{l}{\|}_{2}^{2}\}\leq \rho_{\max}\;l=1,\ldots ,L,$$

where the maximum power $\rho_{\max}$ (uniform across all APs) represents the upper transmit power threshold for each AP. The signals received at UE *k* from all linked APs are labeled as follows:

(25) $$u_{k}=\sum_{l\in M_{k}}h_{kl}^{H}x_{l}+n_{k},$$

where $n_{k}$ is the Gaussian noise generated during data transmission satisfying $n_{k}∼N_{C}\left(0,\sigma^{2}\right)$, and $\sigma^{2}$ denotes the power of the noise. The achievable SE for UE *k* is as follows:

(26) $${\mathrm{SE}}_{k}=\frac{\tau_{d}}{\tau_{c}}\log_{2}\left(1+{\mathrm{SINR}}_{k}\right),$$

where ${\mathrm{SINR}}_{k}$ represents the ratio of the signal to the interference and noise for UE *k*.

### 4.1. Application in Large Scale Fading Precoding

The LSFP scheme employs a two-layer approach for downlink precoding. Firstly, the CPU selects the LSFP vector $b_{k}={\left[b_{k1},\ldots ,b_{kL}\right]}^{T}$ to process the original data. The LSFP vector for UE *k* is as follows:

(27) $$b_{k}=\sqrt{\rho_{k}}{\bar{b}}_{k},$$

where $\rho_{k}$ denotes the total power allocated to UE *k*, and there are multiple choices for $\rho_{k}$, such as the modified fractional power allocation (MFPA) strategy [20]. ${\bar{b}}_{k}$ denotes the CPU-chosen LSFP vector for UE *k* before power optimization, and using the duality of UL-DL, we have ${\bar{b}}_{k}={{\tilde{e}}_{k}}^{*}$. At the end of the first layer of precoding, the signal sent to UE *k* from AP *l* is expressed as follows:

(28) $$\varsigma_{kl}=b_{kl}^{*}\varsigma_{k}.$$

The CPU then transmits all UEs’ precoded data signals $\left\{\varsigma_{il}:i=1,\ldots ,K\right\}$ to AP *l*.

After receiving the signal from the CPU, the AP *l* utilizes the local precoding vectors, such as the scalable LP-MMSE [10] scheme, as follows:

(29) $${\tilde{w}}_{kl}^{\mathrm{LP}-\mathrm{MMSE}}=p_{k}{\left(\sum_{i\in P_{l}}p_{i}\left({\hat{h}}_{il}{\hat{h}}_{il}^{H}+C_{il}\right)+\sigma^{2}I_{N}\right)}^{-1}{\hat{h}}_{kl}$$

to preprocess the DL signal $\left\{\varsigma_{il}:i=1,\ldots ,K\right\}$ using its understanding of the local channel information. Since the LSFP has an additional layer of precoding, UE *k*’s effective precoding vector at AP *l* is denoted as $w_{kl}={\tilde{w}}_{kl}b_{kl}^{*}$, and the final precoding result at AP *l* in Equation (22) becomes as follows:

(30) $$x_{l}^{\prime }=\sum_{i=1}^{K}D_{il}w_{il}\varsigma_{i}=\sum_{i\in P_{l}}{\tilde{w}}_{il}b_{il}^{*}\varsigma_{i}.$$

In Section 3, we can directly compute UE *k*’s LSFD vector using uplink data. By applying UL-DL duality and noting that ${\tilde{w}}_{kl}=v_{kl}$, we can apply UL LSFD for DL LSFP. Therefore, the LSFP vector ${\bar{b}}_{k}$ before power control can be obtained from Equation (21) as follows:

(31) $${\bar{b}}_{k}={\tilde{e}}_{k}^{*}={\left({\hat{F}}_{k}^{-1}{\hat{f}}_{k}\right)}^{*}.$$

Given the parameter ${\bar{b}}_{k}$, the CPU can allocate power under the constraints of Equation (24) and finally compute the LSFP vector $b_{k}$. Since this process does not require every AP to transmit the local CSI to the CPU, there is no fronthaul overhead involved in this process.

### 4.2. Application in Fractional Power Allocation

In fully distributed DL data transmission, let $\rho_{kl}$ be the power control factor assigned to UE *k* by AP *l*. Then, UE *k*’s effective precoding vector locally at AP *l* is as follows:

(32) $$w_{kl}=\sqrt{\rho_{kl}}{\bar{w}}_{kl}$$

where ${\bar{w}}_{kl}\in C^{N}$ satisfies the following:

(33) $$E\{\|{\bar{w}}_{kl}{\|}_{2}^{2}\}=E\left\{∥{\tilde{w}}_{kl}/\sqrt{E\{\|{\tilde{w}}_{kl}{\|}_{2}^{2}\}}{∥}_{2}^{2}\right\}=1$$

where ${\tilde{w}}_{kl}=v_{kl}$ reuses the UL combining vector $v_{kl}$ for DL precoding, e.g., the scalable LP-MMSE scheme in Equation (29). Under distributed operation, the SINR of UE *k* is as follows:

(34) $${\mathrm{SINR}}_{k}=\frac{{\left|\sum_{l=1}^{L}E\left\{h_{kl}^{H}\sqrt{\rho_{kl}}D_{kl}{\bar{w}}_{kl}\right\}\right|}^{2}}{\sum_{i=1}^{K}E\left\{{\left|\sum_{l=1}^{L}h_{kl}^{H}\sqrt{\rho_{il}}D_{il}{\bar{w}}_{il}\right|}^{2}\right\}-{\left|\sum_{l=1}^{L}E\left\{h_{kl}^{H}\sqrt{\rho_{kl}}D_{kl}{\bar{w}}_{kl}\right\}\right|}^{2}+\sigma^{2}}$$

*Proof:* It uses the same method as in [22], Th. 4.4, but applies it to the signal model given in Equation (25).

In distributed downlink power control, our goal is to find a suitable power allocation coefficient $\rho_{kl}$ to enhance the system’s spectral efficiency while adhering to the constraints set by Equation (24). In [20], the author proposed the generalized fractional power allocation (GFPA) algorithm by finding the equivalent large-scale fading channel factor. Under the GFPA algorithm, $\rho_{kl}$ is set to correspond to the following:

(35) $$\begin{array}{c} \rho_{kl}=\rho_{\max}\frac{{\tilde{\chi }}_{kl}}{{\left(\sum_{\ell \in M_{k}}{\tilde{\chi }}_{k\ell }\right)}^{\alpha }{\left(\sum_{i\in P_{l}}\frac{{\tilde{\chi }}_{il}}{{\left(\sum_{\ell \in M_{i}}{\tilde{\chi }}_{i\ell }\right)}^{\alpha }}\right)}^{\gamma }}, \end{array}$$

where indices α∈[0,1], and γ∈[0.4,1.6]. Note that

(36) $$\begin{array}{c} {\tilde{\chi }}_{kl}=E\left\{h_{kl}^{H}D_{kl}{\tilde{w}}_{kl}\right\}/N \end{array}$$

is an equivalent large-scale fading channel factor proposed by GFPA. According to Equation (35), the calculation of $\rho_{kl}$ requires the CSI from other APs. Since the APs are mutually independent in a distributed operation, this necessitates the CPU to integrate the statistical CSI from each AP, which undoubtedly introduces additional fronthaul overhead.

To solve this issue, we can apply the approach presented in Section 3 to blindly estimate the required CSI of the GFPA via uplink data. When uplink data are transmitted at power $\left\{p_{k}=1,k=1,\ldots ,K\right\}$, using ${\tilde{w}}_{kl}=v_{kl}$ and Equation (14), then, Equation (36) can be rewritten as follows:

(37) $${\tilde{\chi }}_{kl}=E\left\{h_{kl}^{H}D_{kl}{\tilde{w}}_{kl}\right\}/N={\left(E\{{\hat{s}}_{kl}s_{k}^{*}\}\right)}^{*}/N$$

where $E\{{\hat{s}}_{kl}s_{k}^{*}\}$ can be calculated by Equation (19) and Equation (20).

The GFPA algorithm in Equation (36) requires the local CSI to be sent to the CPU at each AP for power control, while Equation (37) shows that we can use the UL data signal to blindly estimate the equivalent large-scale fading channel factor in Equation (36), which avoids the fronthaul transmission overhead of CSI.

### 4.3. CSI Overhead Evaluation

In a cell-free mMIMO system, the APs interact with the CPU via the fronthaul link. The computation of statistical CSI requires each AP to transmit its local channel knowledge to the CPU for integration, which incurs a large amount of forward overhead. In addition to the transmission overhead of the data signals, Table 1 provides a comparison of the additional CSI overhead incurred by the schemes discussed in this paper.

When directly using the partial CSI feedback (PCSIF) in Equation (9) to compute the LSFD vector, each AP *l* needs to send $E\left\{v_{kl}^{H}D_{kl}h_{il}\right\}$ for $k\in P_{l}$, $i\in L_{k}$; $E\left\{{\left|v_{kl}^{H}D_{kl}h_{il}\right|}^{2}\right\}$ for $k\in P_{l}$, $i\in L_{k}$; and $E\left\{{∥v_{kl}D_{kl}∥}_{2}^{2}\right\}$ for $k\in P_{l}$ to the CPU, where each AP does not know the true value of the channel vector $h_{il}$ but can substitute it with the estimated value ${\hat{h}}_{il}$. Therefore, each AP needs to send $\left|P_{l}\right|\left(3\left|L_{k}\right|+1\right)/2$ ($\left|P_{l}\right|$, $\left|L_{k}\right|$ denotes the cardinality of the set $P_{l}$ and $L_{k}$, respectively) complex scalars to the CPU.

When using the scalable statistical CSI estimation (CSIE-S) method described in Equation (21) to calculate the LSFD vector, there is no additional CSI overhead in the fronthaul link because it relies on UL data for estimation. When the statistical CSI estimation (CSIE) scheme from the literature [18] is used, there is no CSI overhead involved either, as it also estimates the LSFD vector using uplink data. However, CSIE is not scalable because it is derived based on Equation (6).

For UE *k*, if the GFPA algorithm in Equation (35) is used to calculate the power control coefficients, each AP *l* needs to send the equivalent large-scale fading channel factors ${\tilde{\chi }}_{kl}$ for all $\left|P_{l}\right|$ UEs it serves to the CPU. For the proposed estimate-based GFPA (GFPA-E) algorithm that uses Equation (37) to estimate ${\tilde{\chi }}_{kl}$ on the CPU using UL data, there is no need for the CSI overhead involved in the fronthaul link.

## 5. Performance Analysis

We use MATLAB-R2021b simulations to evaluate the effectiveness of the scheme presented in this work. We configured L=100 APs and K=50 UEs uniformly distributed over an area. Each AP boasts N=4 antennas, while each UE relies on just one antenna. The maximum DL transmission power is $\rho_{\max}=1000\;\mathrm{mW}$, and the channel bandwidth is 20 MHz. We consider two different scenarios as shown in Table 2.

**Figure 2:**
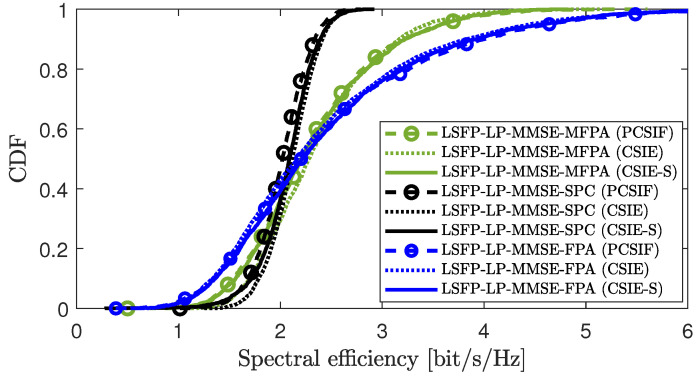
CDF comparison of downlink SE per UE among CSIE-S, CSIE, and PCSIF under different algorithms.

Figure 2 compares the cumulative distribution function (CDF) curves of the DL SE per UE corresponding to different power control algorithms under LSFP for CSIE-S, CSIE, and PCSIF in scenario 1. We consider the LP-MMSE precoding and three different power control strategies as listed in scenario 1 of Table 2. it is evident that compared to the traditional non-scalable CSIE, the proposed CSIE-S method can achieve nearly the same SE performance under various power allocation algorithms while ensuring system scalability. Compared to PCSIF, CSIE-S avoids fronthaul overhead and achieves nearly the same SE performance, even attaining higher SE gains; for example, under the SPC power control scheme, CSIE-S significantly outperforms PCSIF. These results demonstrate the effectiveness and practicality of the CSIE-S method for DL LSFP.

**Figure 3:**
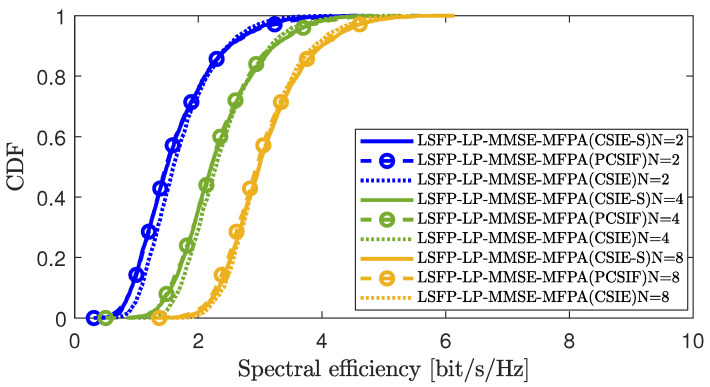
CDF comparison of downlink SE per UE among CSIE-S, CSIE, and PCSIF at different *N*.

Figure 3 compares the CDF curves corresponding to the downlink SE per UE in CSIE-S, CSIE, and PCSIF under the MFPA algorithm in scenario 1. We set up the following three different AP antenna configuration schemes in the simulation: N=2,4, and 8, respectively. The simulation results show that, with the continuous increase of antenna deployment *N*, the system’s spectral efficiency progressively improves. This improvement is attributed to the spatial diversity and multiplexing gain provided by numerous antennas. Multiple antenna arrays can transmit directionally to each UE and receive directionally from them. The multiplexing gain permits the system to send multiple data streams via different antennas simultaneously within the same frequency band, dramatically increasing the system’s capacity and SE. This improves the accuracy of signal detection to a certain extent, and it effectively reduces the interference between UEs. It is essential to emphasize that, regardless of the increase or decrease in the number of antennas, the scalable CSIE-S scheme has always maintained nearly similar performance to CSIE and PCSIF.

In the process of calculating statistical CSI, the complexity of the algorithm mainly arises from matrix inversion and the number of UEs involved. The complexity of CSIE and CSIE-S is summarized in Table 3, using the number of complex multiplications. Here, $\left|L_{k}\right|$ and $\left|M_{k}\right|$ represent the cardinality of the sets $L_{k}$ and $M_{k}$, respectively. Since the number of antennas *N* equipped at each AP is limited, the complexity of CSIE gradually becomes unmanageable as the number of UEs and APs in the area increases. However, the complexity of CSIE-S only depends on the sets $L_{k}$ and $M_{k}$. When $\left|L_{k}\right|$ and $\left|M_{k}\right|$ are constants, regardless of how the number of UEs and APs increases, CSIE-S maintains a finite complexity. Therefore, CSIE-S is more adaptable to large networks with a significant number of UEs and APs.

**Figure 4:**
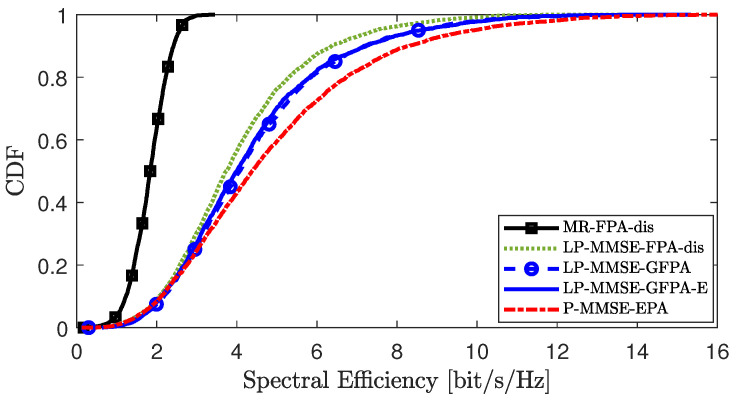
CDF comparison of downlink SE per UE between GFPA and GFPA-E under LP-MMSE precoding.

**Figure 5:**
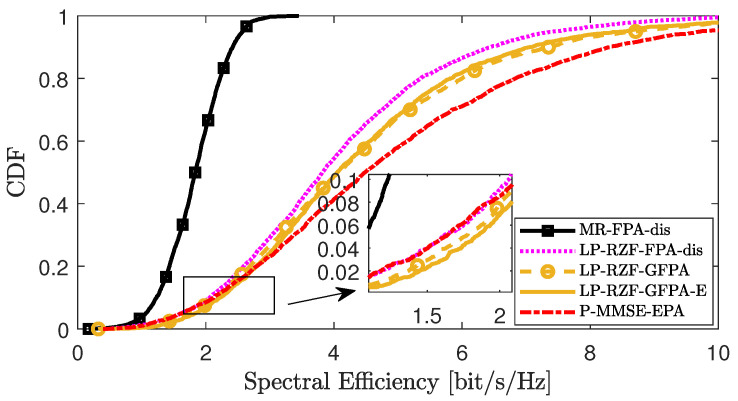
CDF comparison of downlink SE per UE between GFPA and GFPA-E under LP-RZF precoding.

**Figure 6:**
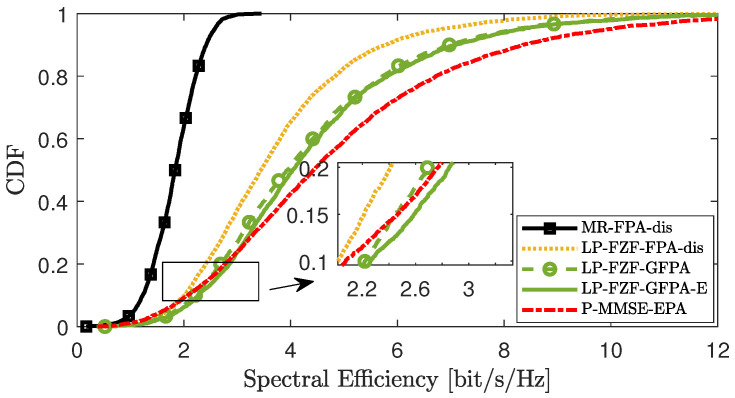
CDF comparison of downlink SE per UE between GFPA and GFPA-E under LP-FZF precoding.

Figure 4, Figure 5 and Figure 6 compare the CDF curves corresponding to the downlink SE per UE under different distributed precoding schemes in scenario 2. We compared the performance of the GFPA-E algorithm proposed in this paper with the existing FPA-dis algorithm and the GFPA algorithm, where the parameters are all set to α=0.8 and γ=1.6. In addition, the MR [10] scheme with the FPA-dis algorithm provides the lower performance limit, while the partial MMSE (P-MMSE) [10] scheme with centralized equal power allocation (P-MMSE-EPA) provides the upper performance limit for the system. The results show that FPA-dis performs poorly. Among the three different precoding schemes, the proposed GFPA-E scheme achieves performance almost comparable to GFPA, and it even achieves higher SE gains at certain points (for example, in the lower tail portions of the CDF curves in Figure 5 and Figure 6). It is important to emphasize that GFPA-E avoids the additional fronthaul CSI overhead, as we derived, since GFPA-E relies on UL data to compute the equivalent large-scale fading channel factor involved in GFPA.

**Figure 7:**
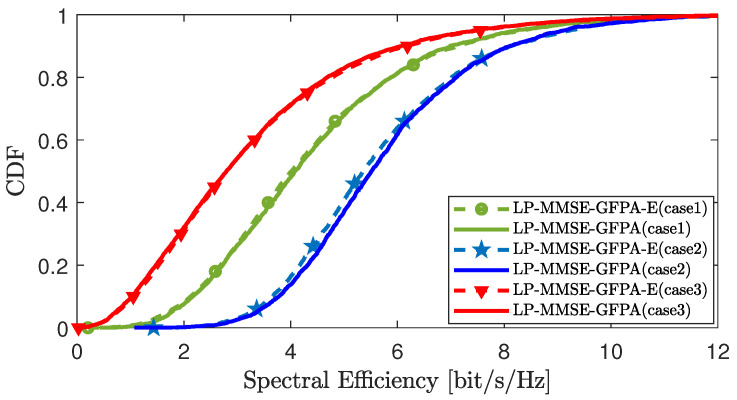
CDF comparison of downlink SE per UE between GFPA and GFPA-E in different scenarios.

Figure 7 compares the CDF curves corresponding to the downlink SE per UE under the GFPA algorithm and the GFPA-E algorithm in scenario 2, where the parameters are set to α=0.8 and γ=1.6. We compared the following three different cases: Case 1, L=100,N=4,K=50; Case 2, L=200, N=4, K=50; and Case 3, L=100, N=4, K=100. The simulation results show that, with the continuous expansion of mobile UEs’ scale in the region, the SE performance of the GFPA and GFPA-E algorithms exhibits the typical multiuser degradation characteristics. This is mainly due to the fact that having more UEs sharing pilot resources exacerbates pilot contamination, thereby reducing channel estimation accuracy. On the contrary, the rise in the quantity of APs notably enhances the system’s performance, as more APs provide greater spatial diversity gain, which effectively suppresses inter-user interference. In addition, regardless of how the scale of UEs and APs changes, the GFPA-E solution achieves nearly similar performance to the GFPA.

**Figure 8:**
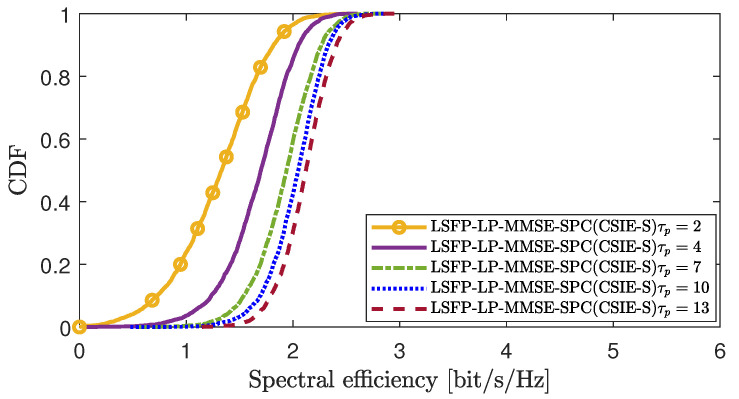
CDF of the DL SE per UE in CSIE-S under different channel estimation errors.

**Figure 9:**
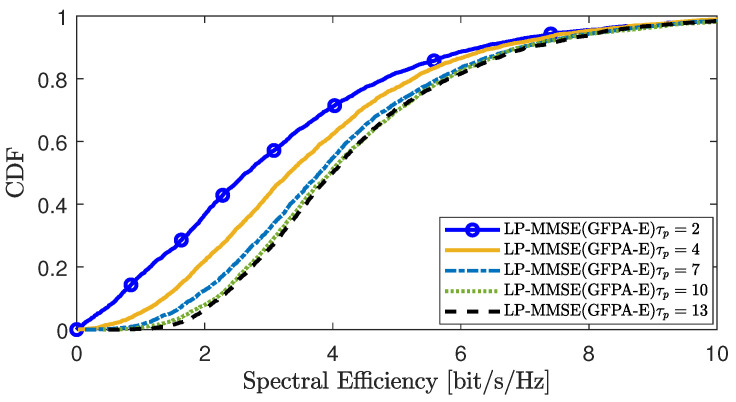
CDF of the DL SE per UE in GFPA-E under different channel estimation errors.

Figure 8 and Figure 9 analyze the impact of pilot contamination or imperfect channel estimation on the performance of the proposed scheme in scenario 1 and scenario 2, respectively. A larger value of $\tau_{p}$ indicates more abundant pilot resources, resulting in reduced pilot contamination and smaller channel estimation errors. From the figures, we can see that when the pilot resource $\tau_{p}$ is very small, different UEs share the same pilot, leading to pilot contamination and inaccurate channel estimation, which degrades the overall performance of the system. As the pilot resource $\tau_{p}$ gradually increases, the pilot contamination situation improves, enhancing the accuracy of channel estimation and thereby improving the system’s performance.

**Figure 10:**
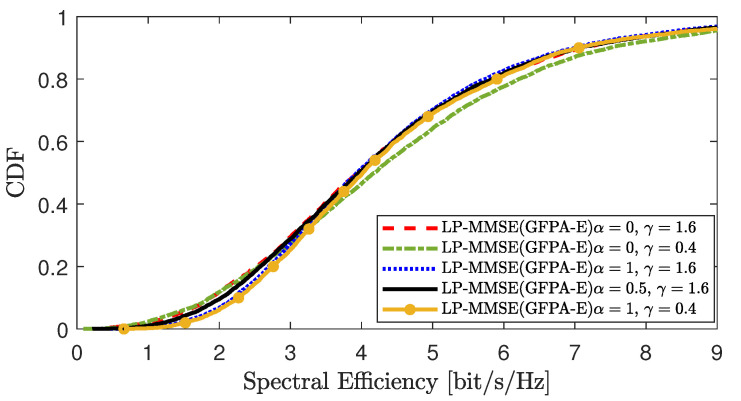
Comparison of DL SE per UE when different parameters are selected for GFPA-E.

Figure 10 compares the impact on the DL SE per UE when different parameters are selected for GFPA-E in scenario 2. Parameters α and γ are introduced to provide flexibility, allowing the system to balance between performance maximization and fairness. As shown in the figure, when α=0, UEs with poor channel conditions (i.e., the lower tail of the CDF curve) fail to receive effective power allocation, resulting in degraded performance for weak UEs. As α gradually increases, the performance of weak UEs gradually improves; and when α=1, the rise of the CDF curve is significantly faster, indicating improved fairness across the system. Under the same conditions, a larger γ value is more favorable to system fairness, while a smaller γ tends to allocate more power to UEs with better channel conditions. However, the overall system performance seems to be insensitive to the choice of parameters α and γ. As long as the values are taken within a reasonable interval, the performance of GFPA-E is stable in different scenarios. This indicates that the method is robust and easy to use in terms of practical parameter settings.

**Figure 11:**
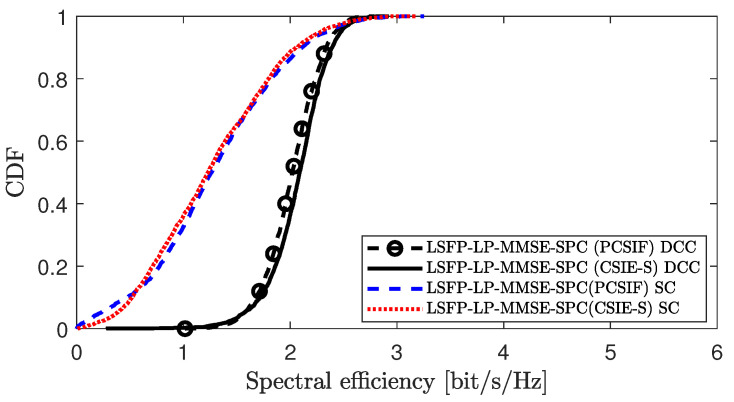
Performance comparison of CSIE-S and PCSIF under different clustering strategies.

Figure 11 compares the performance of the proposed CSIE-S method with the conventional PCSIF method under different clustering strategies in scenario 1. To comprehensively evaluate the adaptability and robustness of the proposed algorithm across various clustering approaches, we consider not only the user-centric dynamic cooperative clustering (DCC) strategy proposed in [10] but also introduce another suboptimal clustering (SC) method, as proposed in [24]. In this method, each UE selects a fixed number of APs with the strongest channel conditions as its serving APs, without considering the effects of pilot contamination. Simulation results show that under both DCC and SC strategies, the CSIE-S method achieves comparable SE performance to the PCSIF scheme. However, the performance of the algorithm under the DCC strategy is significantly better than that under SC. This demonstrates that the CSIE-S method is not only applicable to DCC but also compatible with other clustering strategies, but its performance may vary slightly depending on the specific clustering method employed.

## 6. Conclusions

In cell-free massive MIMO systems, the existing statistical CSI acquisition methods are not scalable in large-scale networks. Therefore, in this paper, we propose a scalable statistical CSI blind estimation method (CSIE-S) based on UL data signals. We demonstrate that the proposed method can be used to obtain the required statistical CSI for LSFP and distributed power control schemes in user-centric cell-free networks. In the LSFP scenario, the CSIE-S scheme not only greatly reduces the CSI fronthaul overhead, but it also achieves spectral efficiency comparable to that of the traditional CSI acquisition scheme while ensuring scalability, which is an important feature for practical implementation. In the fully distributed DL precoding scenario, the proposed method provides a scalable solution to estimate the required statistical CSI for power control strategies such as the GFPA scheme, thereby eliminating the fronthaul overhead for statistical CSI exchange while achieving nearly the same SE.

## Figures and Tables

**Table 1:** Comparison of statistical CSI overhead for different schemes.

Scheme	CSI Overhead
CSIF [18]	K(3K+1)/2
PCSIF	$\left|P_{l}\right|\left(3\left|L_{k}\right|+1\right)/2$
CSIE [18]	0
CSIE-S	0
GFPA [20]	$|P_{l}|$
GFPA-E	0

**Table 2:** Two different scenarios.

Connotation	Scenario 1	Scenario 2
Area size	$1\times 1\;{\mathrm{km}}^{2}$	$2\times 2\;{\mathrm{km}}^{2}$
Pilot training	$\tau_{p}=10$	$\tau_{p}=10$
Uplink data	$\tau_{u}=100$	$\tau_{u}=190$
Downlink data	$\tau_{d}=90$	$\tau_{d}=190$
Local precoding scheme	LP-MMSE [10]	LP-MMSE, LP-RZF [23], LP-FZF [14]
Power control scheme	MFPA [20], FPA [23], SPC [9]	FPA-dis [19], GFPA [20]

**Table 3:** Comparison of the complexity of different schemes.

Scheme	Complexity
CSIE [18]	$O\left(L\left(N+1\right)+KL^{2}+L^{3}\right)$
CSIE-S	$O\left(\left|M_{k}\right|\left(N+1\right)+\left|L_{k}\right|{\left|M_{k}\right|}^{2}+{\left|M_{k}\right|}^{3}\right)$

## Data Availability

The data presented in this study are available upon request from the corresponding author.
